# CXCL1 is elevated in the urine of bladder cancer patients

**DOI:** 10.1186/s40064-015-1393-9

**Published:** 2015-10-15

**Authors:** Andre Burnier, Yoshiko Shimizu, Yunfeng Dai, Masakazu Nakashima, Yoshiyuki Matsui, Osamu Ogawa, Charles J. Rosser, Hideki Furuya

**Affiliations:** John A. Burns School of Medicine, University of Hawaii, Honolulu, HI 96813 USA; Clinical and Translational Research Program, University of Hawaii Cancer Center, 701 Ilalo St, Honolulu, HI 96813 USA; Department of Molecular Biosciences and Bioengineering, University of Hawaii at Manoa, Honolulu, HI 96822 USA; Department of Biostatistics, The University of Florida, Gainesville, FL 32610 USA; Department of Urology, Kansai Electric Power Hospital, Osaka, Japan; Department of Urology, Graduate School of Medicine, Graduate School of Pharmaceutical Sciences, Kyoto University, Kyoto, Japan

**Keywords:** Bladder cancer, CXCL1, Sensitivity, Specificity, Urine

## Abstract

Chemokines, including chemokine (C-X-C motif) ligand 1 (CXCL1), regulate tumor epithelial-stromal interactions that facilitate tumor growth and invasion. Recently, several studies have linked CXCL1 expression to bladder cancer (BCa). In this study, we aimed to determine if increased levels of urinary CXCL1 were found in BCa patients. Voided urines from 86 subjects, cancer subjects (n = 43), non-cancer subjects (n = 43) were analyzed. The protein concentration of CXCL1 was assessed by enzyme-linked immunosorbent assay (ELISA). CXCL1 concentration level was normalized using urinary protein and urinary creatinine concentrations. We used the area under the curve of a receiver operating characteristic (AUROC) to investigate the performance of CXCL1 in detecting BCa. Mean urinary concentrations of CXCL1 were significantly higher in subjects with BCa compared to subjects without BCa (179.8 ± 371.7 pg/mg of creatinine vs. 28.2 ± 71.9 pg/mg, respectively *p* = 0.0009). Urinary CXCL1 possessed a sensitivity of 55.81 %, specificity of 83.72 %, positive predictive value of 77.42 %, negative predictive value of 65.46 %, and an overall accuracy of 69.77 % (AUROC: 0.7015, 95 % CI 0.5903–0.8126). These results indicate that CXCL1 is elevated in BCa when compared to non-cancer subjects, but lacks robustness as a standalone urinary biomarker. Additional studies into CXCL1 may shed more light on the role of CXCL1 in BCa tumorigenesis as well as ramifications of therapeutically targeting CXCL1.

## Background

Chemokines are a family of small peptides that induce directed chemotaxis in nearby responsive cells. Their canonical function is immune and inflammatory reactions, such as allergic disorders, autoimmune diseases, and in viral infections. On the other hand, overexpression and uncontrolled activity of certain chemokines have been implicated in the initiation and progression of several cancers through a variety of mechanisms. Specifically, chronic exposure of cells to a chemokine-rich environment induces accumulation of macrophage and T cell, chronic activation of macrophages, abnormal angiogenesis, and DNA damage because of the presence of reactive oxygen species (Gillitzer and Goebeler 2001). In addition, it is known that chemokines regulate multiple processes associated with tumor growth and progression including primary tumor growth, tumor angiogenesis and development of metastatic disease. Thus, due to these effects, some reports have linked chemokines to more aggressive cancers (Hembruff and Cheng 2009).

One chemokine of interest is chemokine (C-X-C) ligand 1 (CXCL1), also known as growth-regulated oncogene-alpha or melanoma growth stimulatory activity, alpha. Accumulating evidence suggest that CXCL1 is overexpressed in colon, skin and breast cancers (Verbeke et al. 2011; Dhawan and Richmond 2002; Vazquez-Martin et al. 2007; Lerebours et al. 2008). Previously, Kawanishi et al. reported significantly higher urinary CXCL1 concentrations in patients with muscle invasive bladder cancer (MIBC) relative to non-muscle invasive bladder cancer (NMIBC), suggesting CXCL1 as an independent factor for predicting the invasive phenotype (Kawanishi et al. 2008). In an urine-based follow-up study, Nakashima et al. confirmed these results with measuring the concentration of urinary CXCL1 in 175 patients with bladder cancer (BCa) and 30 healthy volunteers (Nakashima et al. 2015). Subsequently, our group demonstrated that CXCL1 protein expression was present in cancerous tissues and entirely absent in benign tissue. Furthermore, CXCL1 immunostaining was significantly higher in high-grade tumors and MIBC relative to low-grade tumors and NMIBC, respectively. Increased CXCL1 immunostaining was similarly associated with reduced disease-specific survival (Miyake et al. 2013).

Herein, we aimed to expand on the above data and determine if increased urinary levels of CXCL1 are found in BCa patients.

## Methods

### Specimen and data collection

Under Institutional Review Board approval by Western Institutional Review Board (Puyallup, WA) and informed consent (IRB #Rosser 2014-1), voided urine samples and associated clinical information were prospectively collected. The urine samples were randomly collected from each subject. Urine cytology was collected at the same time as the urine samples. The study cohort consisted of 43 subjects with no previous history of urothelial carcinoma, gross hematuria, active urinary tract infection, or urolithiasis, and 43 subjects with newly diagnosed primary urothelial carcinoma. Median follow-up was 6 months. This study, which consisted of 86 subjects, adhered to PRoBE (Feng et al. 2013) and STARD criteria (Bossuyt et al. 2004). All subjects were evaluated in the outpatient Urology clinic. Urinalysis and urinary cytology were performed on all subjects. Furthermore, in our cancer group, axial imaging of the abdomen and pelvis and cystoscopy were performed, and urothelial carcinoma was confirmed by histological examination of excised tissue.

### Specimen processing and analysis

Prior to any type of therapeutic intervention, 100 mL of voided urine was obtained from each subject. Voided urine sample was immediately placed into 4 °C refrigerator after collection. Fifty milliliters of urine was sent to the clinical laboratory for urinalysis and voided urinary cytology (VUC). The remaining 50 mL of urine was assigned a unique identifying number before immediate delivery and laboratory processing. The urine sample was transported to the laboratory under refrigerated conditions. Within 4 h of collection, the sample was processed in the laboratory. Each urine sample was centrifuged at 600×*g* 4 °C for 5 min. The supernatant was decanted and aliquoted, and the urinary pellet was snap frozen. Both the supernatant and pellet were stored at −80 °C prior to analysis.

### Assessment of urinary CXCL1, creatinine and protein levels

The level of human CXCL1 (Cat# DGR00 R & D Systems Inc., Minneapolis, USA) was monitored in urine samples using enzyme-linked immunosorbent assays (ELISA). Readers of this assay were blinded as to disease status. The assays were conducted according to the manufacturer’s instructions. Calibration curves were prepared using purified standards. Curve fitting was accomplished by either linear or four-parameter logistic regression following manufacturer’s instructions.

The relatively constant production of creatinine, a non-enzymatic metabolite of creatine, makes urinary creatinine a useful tool for normalizing the levels of other molecules found in urine (Reid et al. 2012). The concentration of urinary creatinine was measured using a commercially available enzymatic assay (Cat# KGE005 R & D Systems Inc., Minneapolis, MN, USA) according to the manufacturer’s instruction. Briefly, urine supernatants were treated with alkaline picrate solution, which yields an orange-red color when in the presence of creatinine. Intensity of the color at 490 nm corresponds to the concentration of creatinine in the sample. Creatinine concentrations of unknown samples were calculated by comparison to a standard curve.

### Data analysis

The association between CXCL1 and BCa was tested using Wilcoxon rank sum test. Nonparametric receiver operating characteristic (ROC) curves were generated in which the value for sensitivity is plotted against false-positive rate (1-specificity). Areas under ROC curves were estimated and compared by Chi-square test. We defined a diagnostic test (positive vs. negative) for BCa using a cutoff threshold for CXCL1. The optimal cutoff (Youden index) was selected to maximize the sum of the sensitivity and specificity (Edgar et al. 2002). The overall accuracy of a biomarker to predict BCa is defined as the percentage of correctly predicted BCa or non-BCa. To assess the independent association of CXCL1 to BCa, logistic regression analysis was performed with BCa status (yes vs. no) as the dependent variable and with gender, age, race, tobacco exposure and CXCL1 concentrations as explanatory variables. Statistical significance in this study was set at *p* < 0.05 and all reported *p* values were two-sided. All analyses were performed with SAS software version 9.4.

## Results

**Fig. 1:**
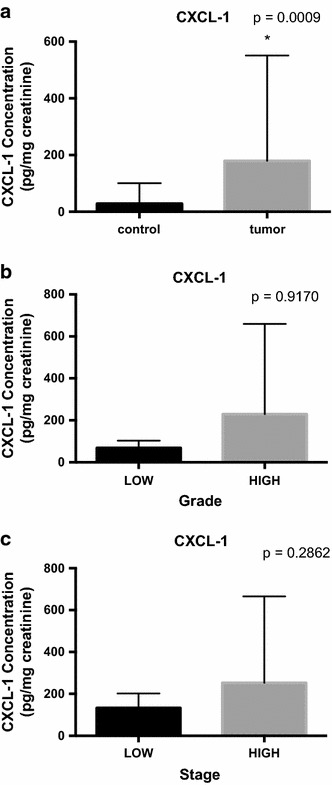
Comparison of urine concentrations of CXCL1 between **a** the cancer and non-cancer groups, **b** low-grade and high-grade BCa and **c** low stage (NMIBC) and high stage BCa (MIBC). Data are normalized to urinary creatinine. Horizontal lines depict median levels. Significance (*p* < *0.05*) was assessed by the Wilcoxon rank sum test

The cohort of 86 subjects consisted of 43 subjects with active BCa and 43 control subjects. Demographic, clinical, and pathologic characteristics of both groups are illustrated in Table 1. In the cancer cohort, VUC had a sensitivity of only 51 %. Due to the unavoidable variability of voided urine with respect to total volume and time within the bladder, CXCL1 was normalized to urinary creatinine. Mean urinary concentrations of CXCL1 were significantly higher in subjects with BCa compared to subjects without BCa (179.8 ± 371.7 pg/mg of creatinine vs. 28.2 ± 71.9 pg/mg, respectively *p* = 0.0009). Though the mean urinary CXCL1 concentrations were elevated in high grade vs. low-grade disease (229.5 ± 430.2 vs. 69.3 ± 112.0 pg/mg, respectively *p* = 0.9170) and high-stage vs. low-stage disease (253.1 ± 412.6 vs. 133.2 ± 345.9 pg/mg, respectively *p* = 0.2862), the results were not significant. ELISA data are depicted in Fig. 1.

**Table 1:** Demographic, clinicopathologic characteristics and concentration of urinary proteins in the study cohort

	Non-cancer (%) N = 43	Cancer (%) N = 43
Median age (range, y)	63 (34–81)	67 (20–89)
Male:female ratio	36:7	36:7
Race		
White	27 (63)	41 (95)
African American	2 (5)	1 (2)
Other	14 (33)	1 (2)
Tobacco use	16 (37)	32 (74)
Suspicious/positive cytology	0 (0)	22 (51)
Median follow-up (range, months)	3.5 (1–49)	6 (1–43)
Clinical stage		
Tis	n/a	0 (0)
Ta	n/a	14 (33)
T1	n/a	11 (26)
T2	n/a	16 (37)
T3/4	n/a	2 (4)
Grade		
Low	n/a	31 (72)
High	n/a	12 (28)

**Fig. 2:**
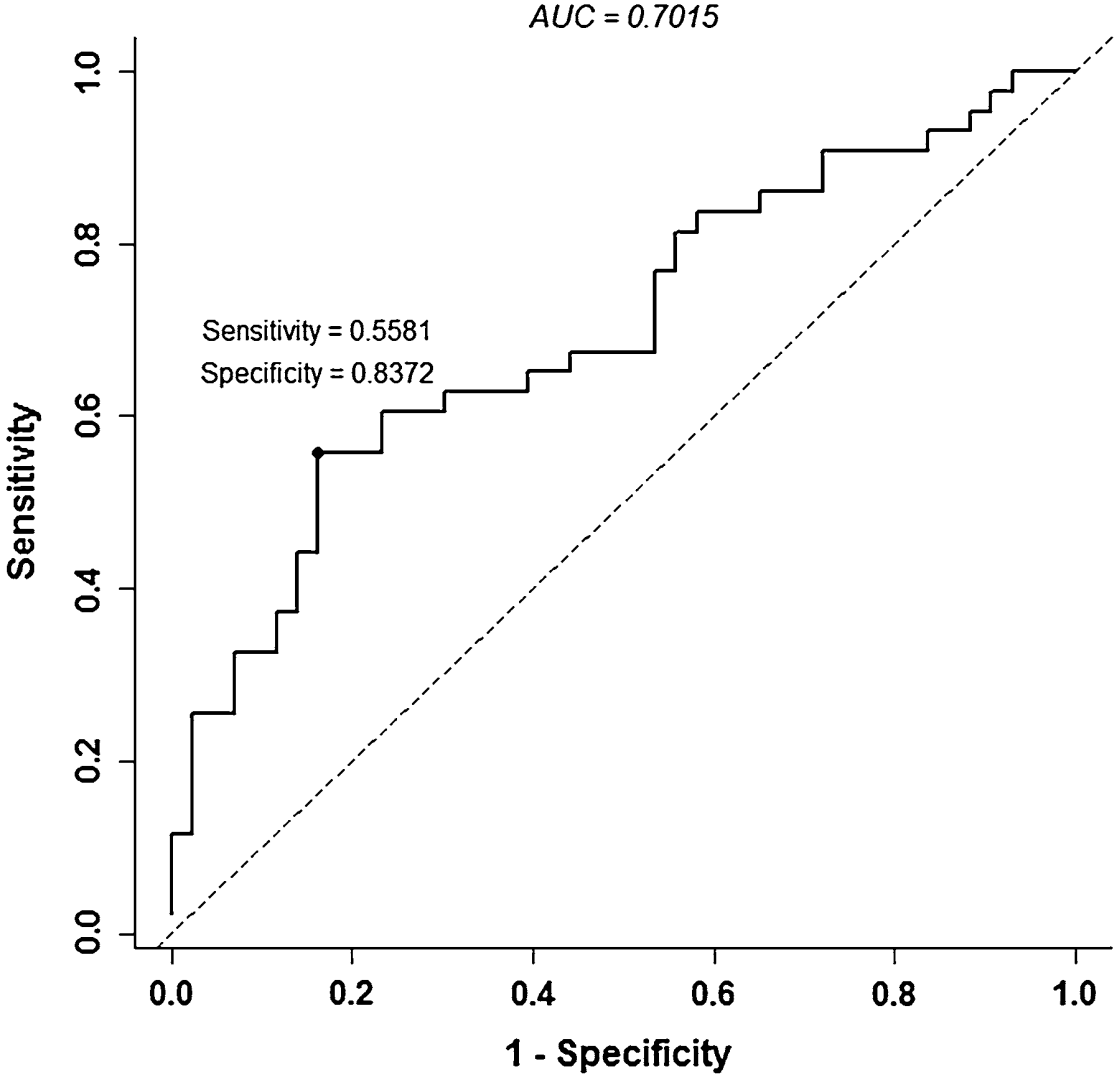
Receiver operating characteristic (ROC) curves for urinary CXCL1. Based on the area under the ROC curve (AUROC), Youden Index cutoff values that maximized the sum of sensitivity and specificity were determined for each biomarker

The ability of CXCL1 to predict the presence of BCa in voided urines was analyzed using nonparametric ROC analyses and the area under the ROC curve (AUROC), according to National Cancer Institute guidelines (Pepe et al. 2008). We determined the Youden Index cutoff values, which maximize the sum of sensitivity and specificity. Using the Youden Index cutoff value, urinary CXCL1 provided a sensitivity of 55.81 %, specificity of 83.72 %, positive predictive value of 77.42 %, negative predictive value of 65.46 % with an overall accuracy of 69.77 % (AUROC: 0.7015, 95 % CI 0.5903–0.8126, Fig. 2).

## Discussion

Cancer of the urinary bladder is among the five most common malignancies worldwide (Siegel et al. 2014). At presentation, more than 80 % of bladder tumors are NMIBC (Ta, T1 or Tis), which harbor a 5-year survival rate of approximately 94 %, however, approximately 70 % of patients with these lesions develop tumor recurrence within 2 years of initial diagnosis (Brausi et al. 2011). The recurrence phenomenon of NMIBC makes it one of the most prevalent cancers world-wide (in America it is second only to colorectal cancer) and is, therefore, a great burden to our healthcare system (Surveillance, Epidemiology, and End Results Program 2013). Then at presentation, approximately 20 % of bladder tumors are MIBC (T2-T4), which carries at best a 50 % 5-year survival rate, or metastatic, which portends a dismal 5-year survival rate <20 % (Witjes et al. 2014). Thus, we continue to search for means to enable early detection of BCa, ideally through non-invasive urine-based analysis.

In this current report, we describe the analysis of urinary CXCL1 in a cohort of 86 subjects using a commercial ELISA assay. Urinary protein concentration of CXCL1 was significantly associated with BCa compared to controls (179.8 ± 371.7 vs. 28.2 ± 71.9 pg/mg, respectively *p* = 0.0009). However, its diagnostic capability as a standalone biomarker, sensitivity of 55.81 %, specificity of 83.72 %, positive predictive value of 77.42 %, negative predictive value of 65.46 % with an overall accuracy of 69.77 %, was less than ideal.

CXCL1 has not been widely reported on in the BCa literature, although a related chemokine, interleukin 8 (IL8), has been extensively studied (Reis et al. 2012; Leibovici et al. 2005; Escudero-Lourdes et al. 2012; Black and Dinney 2007). Oncomine analysis of datasets posted by Sanchez-Carbayo et al*. (*2002), Dyrskjot et al*. (*2003), Lee et al. (2010) demonstrated significant elevation of CXCL1 mRNA levels in human BCa tissues, specifically higher CXCL1 mRNA levels in MIBC compared to NMIBC. The data stand to further substantiate our previous reported findings that CXCL1 protein was noted to be significantly increased in high-stage compared to low-stage and high-grade compared to low-grade BCa tissues (Miyake et al. 2013). In this study, there were no significant differences in urinary CXCL1 levels between high-stage and low-stage, and high-grade and low-grade. This may be due to different samples, tumor and urine. Since previous studies employed BCa tissues, it is considered that CXCL1 levels directly reflect BCa stage and grade. However, we analyzed CXCL1 levels in voided urine. Previous studies have shown that urinary protein analysis is a tool for detecting genitourinary diseases including prostate cancer, renal cell carcinoma and BCa (Adachi et al. 2006), meaning that urinary CXCL1 levels might be affected by other factors in addition to BCa.

Unfortunately, CXCL1 expression is not unique to BCa. Several benign inflammatory bladder disorders are noted to express and secrete higher levels of CXCL1 compared to normal bladder tissue (Tyagi et al. 2010; Zhao et al. 2015). Such benign conditions can then adversely affect the specificity of CXCL1, further limiting its role as a single diagnostic biomarker. However, since CXCL1 is overexpressed and secreted in variety of disease states, thought should be given to its potential as a therapeutic target.

We recognize that our study has several limitations. First, our analyses were performed on processed, banked urines. These urines were centrifuged and separated into cellular pellet and supernatant prior to storage at −80 °C. It is feasible that freshly voided urine samples may provide different results, and it is fresh urine that would be the material used for point-of-care assays. Second, it is uncertain how the protein composition of the urine supernatant may change during frozen storage. The number of freeze–thaw cycles was kept to 1–2 by dividing the urine supernatant into multiple small aliquots. Next, we are a tertiary care facility that is preferentially referred high grade, higher stage disease, which is reflected in our cohort. Lastly, we believe that a more robust diagnostic approach would encompass a diagnostic signature composed of numerous biomarkers, as we previously published (Chen et al. 2014; Rosser et al. 2013, 2014).

## Conclusions

We have confirmed that urinary levels of CXCL1 were elevated in a population of 43 BCa patients relative to 43 control subjects. Though CXCL1 may lack robustness as a sole diagnostic biomarker, it may be a viable therapeutic target in the future.

## Abbreviations

CXCL1: chemokine (C-X-C motif) ligand 1
BCa: bladder cancer
MIBC: muscle invasive bladder cancer
NMIBC: non-muscle invasive bladder cancer
AUROC: area under the curve of a receiver operating characteristic
ELISA: enzyme-linked immunosorbent assay
AUC: area under the curve
CI: confidence interval
OR: odds ratio
ROC: receiver operating characteristics
VUC: voided urinary cytology
IL8: interleukin 8

## References

[CR1] Adachi J, Kumar C, Zhang Y, Olsen JV, Mann M (2006). The human urinary proteome contains more than 1500 proteins, including a large proportion of membrane proteins. Genome Biol.

[CR2] Black PC, Dinney CP (2007). Bladder cancer angiogenesis and metastasis–translation from murine model to clinical trial. Cancer metastasis Rev.

[CR3] Bossuyt PM, Reitsma JB, Bruns DE, Gatsonis CA, Glasziou PP, Irwig LM, Lijmer JG, Moher D, Rennie D, de Vet HC, Group S (2004). Towards complete and accurate reporting of studies of diagnostic accuracy: the STARD initiative. Fam Pract.

[CR4] Brausi M, Witjes JA, Lamm D, Persad R, Palou J, Colombel M, Buckley R, Soloway M, Akaza H, Bohle A (2011). A review of current guidelines and best practice recommendations for the management of nonmuscle invasive bladder cancer by the International Bladder Cancer Group. J Urol.

[CR5] Chen LM, Chang M, Dai Y, Chai KX, Dyrskjot L, Sanchez-Carbayo M, Szarvas T, Zwarthoff EC, Lokeshwar V, Jeronimo C, Parker AS, Ross S, Borre M, Orntoft TF, Jaeger T, Beukers W, Lopez LE, Henrique R, Young PR, Urquidi V, Goodison S, Rosser CJ (2014). External validation of a multiplex urinary protein panel for the detection of bladder cancer in a multicenter cohort. Cancer Epidemiol Biomark Prev Publ Am Assoc Cancer Res Cosponsored Am Soc Prev Oncol.

[CR6] Dhawan P, Richmond A (2002). Role of CXCL1 in tumorigenesis of melanoma. J Leukoc Biol.

[CR7] Dyrskjot L, Thykjaer T, Kruhoffer M, Jensen JL, Marcussen N, Hamilton-Dutoit S, Wolf H, Orntoft TF (2003). Identifying distinct classes of bladder carcinoma using microarrays. Nat Genet.

[CR8] Edgar R, Domrachev M, Lash AE (2002). Gene Expression Omnibus: NCBI gene expression and hybridization array data repository. Nucleic Acid Res.

[CR9] Escudero-Lourdes C, Wu T, Camarillo JM, Gandolfi AJ (2012). Interleukin-8 (IL-8) over-production and autocrine cell activation are key factors in monomethylarsonous acid [MMA(III)]-induced malignant transformation of urothelial cells. Toxicol Appl Pharmacol.

[CR10] Feng Z, Kagan J, Pepe M, Thornquist M, Ann Rinaudo J, Dahlgren J, Krueger K, Zheng Y, Patriotis C, Huang Y, Sorbara L, Thompson I, Srivastava S (2013). The early detection research network’s specimen reference sets: paving the way for rapid evaluation of potential biomarkers. Clin Chem.

[CR11] Gillitzer R, Goebeler M (2001). Chemokines in cutaneous wound healing. J Leukoc Biol.

[CR12] Hembruff SL, Cheng N (2009). Chemokine signaling in cancer: implications on the tumor microenvironment and therapeutic targeting. Cancer Ther.

[CR13] Kawanishi H, Matsui Y, Ito M, Watanabe J, Takahashi T, Nishizawa K, Nishiyama H, Kamoto T, Mikami Y, Tanaka Y, Jung G, Akiyama H, Nobumasa H, Guilford P, Reeve A, Okuno Y, Tsujimoto G, Nakamura E, Ogawa O (2008). Secreted CXCL1 is a potential mediator and marker of the tumor invasion of bladder cancer. Clin Cancer Res.

[CR14] Lee JS, Leem SH, Lee SY, Kim SC, Park ES, Kim SB, Kim SK, Kim YJ, Kim WJ, Chu IS (2010). Expression signature of E2F1 and its associated genes predict superficial to invasive progression of bladder tumors. J Clin Oncol Off J Am Soc Clin Oncol.

[CR15] Leibovici D, Grossman HB, Dinney CP, Millikan RE, Lerner S, Wang Y, Gu J, Dong Q, Wu X (2005). Polymorphisms in inflammation genes and bladder cancer: from initiation to recurrence, progression, and survival. J Clin Oncol Off J Am Soc Clin Oncol.

[CR16] Lerebours F, Vacher S, Andrieu C, Espie M, Marty M, Lidereau R, Bieche I (2008). NF-kappa B genes have a major role in inflammatory breast cancer. BMC Cancer.

[CR17] Miyake M, Lawton A, Goodison S, Urquidi V, Gomes-Giacoia E, Zhang G, Ross S, Kim J, Rosser CJ (2013). Chemokine (C-X-C) ligand 1 (CXCL1) protein expression is increased in aggressive bladder cancers. BMC Cancer.

[CR18] Nakashima M, Matsui Y, Kobayashi T, Saito R, Hatahira S, Kawakami K, Nakamura E, Nishiyama H, Ogawa O (2015). Urine CXCL1 as a biomarker for tumor detection and outcome prediction in bladder cancer. Cancer Biomark.

[CR19] Pepe MS, Feng Z, Janes H, Bossuyt PM, Potter JD (2008). Pivotal evaluation of the accuracy of a biomarker used for classification or prediction: standards for study design. J Natl Cancer Inst.

[CR20] Reid CN, Stevenson M, Abogunrin F, Ruddock MW, Emmert-Streib F, Lamont JV, Williamson KE (2012). Standardization of diagnostic biomarker concentrations in urine: the hematuria caveat. PLoS One.

[CR21] Reis ST, Leite KR, Piovesan LF, Pontes-Junior J, Viana NI, Abe DK, Crippa A, Moura CM, Adonias SP, Srougi M, Dall’Oglio MF (2012). Increased expression of MMP-9 and IL-8 are correlated with poor prognosis of Bladder Cancer. BMC Urol.

[CR22] Rosser CJ, Ross S, Chang M, Dai Y, Mengual L, Zhang G, Kim J, Urquidi V, Alcaraz A, Goodison S (2013). Multiplex protein signature for the detection of bladder cancer in voided urine samples. J urol.

[CR23] Rosser CJ, Chang M, Dai Y, Ross S, Mengual L, Alcaraz A, Goodison S (2014). Urinary protein biomarker panel for the detection of recurrent bladder cancer. Cancer Epidemiol Biomark Prev Publ Am Assoc Cancer Res Cosponsored Am Soc Prev Oncol.

[CR24] Sanchez-Carbayo M, Socci ND, Charytonowicz E, Lu M, Prystowsky M, Childs G, Cordon-Cardo C (2002). Molecular profiling of bladder cancer using cDNA microarrays: defining histogenesis and biological phenotypes. Cancer Res.

[CR25] Siegel R, Ma J, Zou Z, Jemal A (2014). Cancer statistics (2014). CA Cancer J Clin.

[CR26] Surveillance, Epidemiology, and End Results Program (2013). http://www.seer.cancer.gov/csr/1975_2010/. Accessed 16 Dec 2013

[CR27] Tyagi P, Barclay D, Zamora R, Yoshimura N, Peters K, Vodovotz Y, Chancellor M (2010). Urine cytokines suggest an inflammatory response in the overactive bladder: a pilot study. Int Urol Nephrol.

[CR28] Vazquez-Martin A, Colomer R, Menendez JA (2007). Protein array technology to detect HER2 (erbB-2)-induced ‘cytokine signature’ in breast cancer. Eur J Cancer.

[CR29] Verbeke H, Struyf S, Laureys G, Van Damme J (2011). The expression and role of CXC chemokines in colorectal cancer. Cytokine Growth Factor Rev.

[CR30] Witjes JA, Comperat E, Cowan NC, De Santis M, Gakis G, Lebret T, Ribal MJ, Van der Heijden AG, Sherif A, EuropeanAssociation of U (2014). EAU guidelines on muscle-invasive and metastatic bladder cancer: summary of the 2013 guidelines. Eur Urol.

[CR31] Zhao Y, Zhu L, Zhou T, Zhang Q, Shi S, Liu L, Lv J, Zhang H (2015). Urinary CXCL1: a novel predictor of IgA nephropathy progression. PLoS One.

